# Motivations & Experiences of Postgraduate Anatomy Training

**DOI:** 10.15694/mep.2019.000062.1

**Published:** 2019-03-20

**Authors:** James Cameron, Justin Bilszta, Katharine J Reid, Chris Briggs

**Affiliations:** 1 Epworth Hospital; 2 Melbourne Medical School; 3 Faculty of Health

**Keywords:** anatomy, training, undergraduate, education, experiences, qualitative

## Abstract

This article was migrated. The article was marked as recommended.

**Background:** Anatomy teaching at medical schools has undergone significant changes in philosophy, and reduction in content, in recent years. Senior clinicians and speciality training Colleges have raised concerns regarding these changes and questioned their impact on ‘anatomical competence’ and adequacy of training for safe clinical practice. The literature on the perceptions of medical school anatomy teaching among those preparing to enter post-graduate training (i.e. towards a specialist qualification) is sparse.

**Aim:** To assess the motivations for study, and experiences of training, in junior doctors undertaking an intensive post-graduate anatomy training program.

**Methods:** A sample of candidates (13/119, 10%) undertaking the University of Melbourne Graduate Diploma in Surgical Anatomy were recruited for interview. These interviews were recorded, transcribed and then analysed using a combination of thematic and contextual approaches. Key themes were identified and explored.

**Results:** Participant responses fell into two broad categories - motivations for enrolling into the course and their actual experiences of the course. The primary motivation for enrolling into the course was the perceived career requirement to do so, with participants asserting that attending such courses was perceived as mandatory for success in specialty training. Once enrolled, participants valued the teaching and learning and enjoyed the academic pursuit of high-level anatomy study. These benefits, however, were offset by a range of undesirable outcomes associated with undertaking the course. Participants identified the financial cost of the course, the unwillingness of employers to provide rostered study leave and the negative impact on work-life balance as the most significant challenges.

**Conclusions:** Understanding the concerns and expectations of junior doctors preparing for a speciality training program by increasing their anatomy knowledge has implications for both Colleges and medical educators. The participants in this study recognised the limitations in their anatomy knowledge and actively sought additional training at significant financial and personal cost to themselves. This was counterbalanced by the perceived benefits to their career, and an opportunity to enter a specialist training program, by completing additional study.

## Introduction

A knowledge of anatomy has long been considered one of the key aspects of medical training ( Sugand, Abrahams and Khurana, 2010). However, in recent times there has been an upheaval in anatomy instruction ( General Medical Council, 2009), which has led to a swing away from teaching all basic sciences. Instead, anatomy teaching (and bioscience teaching, in general) has attempted to integrate the learning of basic and clinical sciences as disease processes rather than separate entities.

Worldwide, the time available for teaching anatomy has been markedly reduced ( Gogalniceanu, O’Connor and Raftery, 2009; Craig *et al.*, 2010; Hildebrandt, 2010; Bohl and Gest, 2011; Inuwa *et al.*, 2012). Further challenges to teaching anatomy include ongoing efforts to reduce the duration of the basic (i.e. undergraduate/pre-vocational) medical degree; the down-grading of anatomical training in response to changing demands/philosophy of medical school curriculum ( Drake, 2013); a reduction in the availability of dissection facilities ( Papa and Vaccarezza, 2013) and; the loss of experienced teachers of anatomy as academics retire and are not replaced ( Standring, 2009). The best approach to teaching anatomy (as well as the other basic sciences) has also been the subject of significant debate; including what aspects should be taught and the best ways to assess student learning. A schism has developed between a modern approach which favours novel methods of anatomy teaching (e.g. online interactive text and image overlays, virtual and extended reality, anatomy visualization systems, etc.) versus the traditional approach of practical lessons using cadaveric dissection by students ( Patel and Moxham, 2005; Inuwa *et al.*, 2012; Ramsey-Stewart, 2014). These factors highlight the potential for wide variation in the amount, standard and method of anatomical instruction in undergraduate medical programs.

Given these changes, what has been the impact on undergraduate medical students’ ‘anatomical competence’? Do undergraduate medical students have the anatomy knowledge required to enter the workforce as a competent practitioner and to provide safe and effective medical practice? Several research studies have examined undergraduate medical students’ anatomy knowledge and/or perceptions of their knowledge ( Insull, Kejriwal and Blyth, 2006; Burgess and Ramsey-Stewart, 2014; Choi-Lundberg *et al.*, 2015) and have reported conflicting findings. Students have described their factual anatomy knowledge as ‘poor’ ( Burgess and Ramsey-Stewart, 2014), but in other studies students have rated their current anatomy knowledge as adequate to excellent (87% vs 13% who rated their knowledge as inadequate to poor) ( Choi-Lundberg *et al.*, 2015), or adequate to practice medicine safely (52/156, 33%, of respondents) ( Insull, Kejriwal and Blyth, 2006). The caveat to these findings is whether students are in the best position to identify what anatomy they will need to know as a practising clinician? Two studies have also evaluated the views of speciality training program directors, clinical teachers and practising clinicians regarding the adequacy of medical school anatomy education ( Cottam, 1999; Gogalniceanu, O’Connor and Raftery, 2009). In both studies, less than half (43% and 29%, respectively) of those surveyed believed that their trainees had an adequate preparation in anatomy to start their advanced training.

The current evidence base lacks the perspectives of junior doctors contemplating, or about to commence, a speciality training program. Although some researchers have asked junior doctors to consider ‘..how much anatomy is necessary [for clinical practice]..?’ ( Pabst, 2009) or ‘..what is the relevance of all subjects in the undergraduate curriculum for training to become a medical doctor?,..’ ( Pabst and Rothkötter, 1997), only one ( Bohl and Gest, 2011) directly asked junior doctors whether they had received enough anatomy preparation in medical school to enter speciality training. In this study, respondents believed that medical school had not provided sufficient anatomy teaching and they advocated for greater emphasis on teaching anatomy in medical school.

Given that anatomy teaching in undergraduate medical training has reduced and that medical graduates are perceived to be underprepared, postgraduate educational courses have developed to address this gap. Such courses emphasise the key role of dissection and other best-practice teaching methods in acquiring anatomy knowledge. One such course is the Graduate Diploma in Surgical Anatomy ( Briggs, 2014) offered by the University of Melbourne’s Department of Anatomy and Neuroscience ( https://study.unimelb.edu.au/find/courses/graduate/graduate-diploma-in-surgical-anatomy/). Accredited by the Royal Australasian College of Surgeons (RACS), the Diploma is a single semester program comprising a weekly combination of lectures, tutorials and dissection (delivered in 8 hours per week), with a teaching faculty comprising senior clinicians, anatomists and recent course graduates. The course is specifically designed to assist participants preparing to undertake the Surgical Education and Training (SET) Surgical Sciences Examination of RACS. Positions in the course are limited to 120 enrolments per semester. Selection preference is generally given to those in their second and third years of training, although on occasion first-year trainees have been permitted to enrol.

The course comprises four segments (introduction/limbs, spine/head/neck/brain, thorax/abdomen & pelvis/perineum/review); each participant’s program is customised to their area of interest with appropriate dissections. The course also includes exposure to prosected specimens covering all body regions through small-group tutorials, with additional surgically-focused tutorials being provided by specialists. Additional course resources include written and online material and access to the Anatomedia website ( https://anatomedia.com/). Candidates also receive open access to the dissection laboratory, allowing further study outside the formal classroom schedule.

Although a small body of research has evaluated medical students’ perspectives of their medical school anatomy knowledge as well as the views of senior doctors and specialist training program directors, work exploring the perceptions of undergraduate medical school anatomy training among those imminently preparing for post-graduate training (towards a specialist qualification) is sparse. The current study is intended to address this gap in the literature. Specifically, this study sought to explore: (i) the experiences of junior doctors enrolling in a specialist postgraduate anatomy training program; (ii) the motivations for junior doctors to enter a specialist postgraduate anatomy training program and; (iii) the perceptions of junior doctors regarding the benefits of further specialist anatomy training.

## Methods

A qualitative approach was chosen to explore the experiences of diplomates from the 2017 cohort of the Graduate Diploma in Surgical Anatomy.

All students from the cohort were informed of the research project in a presentation and all were invited to participate. Those who agreed to participate undertook a one-hour face-to-face semi-structured interview. To direct interviews, a guide (see Supplementary File) was developed by JC, JB & KR with a series of questions that explored participants’ motivations for enrolling in, and experiences of attending, the course. This guide was used to ensure consistent coverage of the topics and questions. Interviews were held at a mutually convenient time. Written informed consent was collected prior to the interviews and confirmed verbally at the start of each interview session. Participant responses were recorded, transcribed (using an external service) and then qualitatively analysed using NVivo 11.4.1.

Following the interviews, the transcripts were analysed (JC) with dynamic coding to identify any major themes, the intent of the student’s responses or any phrases, words, terms or descriptions that illustrated recurring patterns of experience. Statements were organised into logical sub-themes which were then aggregated into theme clusters. Themes that overlapped or had similar context were merged. The resultant themes clusters were checked (JB & KR) against the original description in each transcript to maximise objectivity and allow refinement, or to highlight relationships between clusters. This process was designed to ensure that the developing analysis was systematic, and the data supported the results. Results are presented to illustrate key components of the student’s experiences.

Approval for this study was obtained through the University of Melbourne’s Department of Medical Education Human Ethics Advisory Group (HREC Approval Number - 1647882.1).

## Results/Analysis

A total of 13 participants were interviewed (12 JC, 1 JB) representing 10% of the cohort (13/115). The median age was 27 years (range: 24-33 years), with seven male and six female participants. The median time from graduation from initial medical training was two years (range: 2-5 years) and all but one of the participants was employed in a metropolitan health service. Of those interviewed, eight aspired to surgical training, three to radiology training and two were undecided.

From the analysis of the participant interviews, two broad themes emerged - motivations for enrolling into the course and the actual experiences of the course - with each major theme yielding a range of subthemes.

### Motivations for Enrolling

#### Career Progression

The positive impact of participation on career progression was a key theme expressed by all participants. This was not just in terms of formal training programs, but also in a more general sense. One candidate phrased this perception very clearly, being told directly by their clinical instructors:

‘..you have to do this anatomy course if you’re interested in doing pretty much anything that involves anatomy..’

Undertaking the course was perceived as offering substantial benefits in terms of successful entry into a postgraduate training program as well as making a good impression with supervisors, resulting in improved references for employment as well as training. A greater range of career options was also identified as a motivation for enrolling. One candidate also noted anatomical knowledge as important even though they had not yet chosen a career path:

‘..I don’t have a very clear career goal, so it doesn’t really matter as much I guess . . . if I decide to go surgical, I guess it is useful., it’s something good for . . .my career, and it’s something good for just progressing down the track . . . ‘cause it’s all relevant . . . I was thinking about doing critical care . . . I’d do a lot of central lines and things . . . and it’s useful in anatomy to know things..”

#### Expected Benefits

Participants reported several expected benefits from completing the Diploma, with the most frequently perceived benefit an improved chance of acceptance into post-graduate training programs for their chosen specialty:

‘..doing a course vs not doing a course . . .my perception would be yes. Any sort of learning is a positive. Ah, training of any sort, particularly surgery, is long and arduous, so the more courses you do, the more training you do, the better..’

Participants also described indirect benefits including a perception that undertaking the course may make a good impression on their senior colleagues, or that the course was something that they did to keep pace in their career progression with their colleagues. Some believed that high achievement in the course would also benefit their future career aspirations. Overall, completing the diploma was regarded as almost mandatory in a highly competitive field and not doing the course was believed to be detrimental to career progression.

Another key aspect of entry to any post-graduate training program is acquiring references from supervising specialists. Applicants for post-graduate training have only a finite exposure to, and opportunity to impress, prospective referees. Paradoxically the Diploma both enhances and detracts from this; the opportunities for participants to make a good impression to senior staff during clinical rotations offset by the realization that attending the course can take them away from their rotations:

‘..I think I would’ve probably been able to get to theatre more and impress a bit more, and sort of. . . I think, I think a couple of times there were. . .my colleagues. . . I didn’t get told this directly, but I got told this indirectly. . . that my colleagues weren’t as happy that I was leaving on time,. . Well actually it was always. . . I was always leaving late from work..’

### Improvements in Knowledge and Exam Performance

All participants strongly believed that their anatomical training in medical school had been inadequate and that they needed additional training to address this gap. Moreover, many felt that importance of anatomy knowledge to clinical practice was also not properly emphasised. Detailed anatomical knowledge was also seen as vital in examination performance, particularly for the highly competitive speciality selection processes. Other participants viewed the anatomy knowledge gained through the course as relevant to a range of other examinations they may encounter in their future careers.

Participants often referred to the recommendations of previous graduates of the course in describing the knowledge that they had gained in completing this additional anatomy training:

‘..seeing what my previous colleagues had done, and the amount of knowledge they had gained from it. The fact that they were then able to teach me a lot of. . so, a lot of what I previously hadn’t known, and just seeing how ready they were for the surgical science exam..’

### Experiences of The Course

#### Barriers to Attendance: Cost, Time, Leave & Rostering and Travel

The financial burden (in 2017, course fees were set at AUS$18,000) of the program was a significant barrier identified by participants and most felt that it be cost-prohibitive were it not necessary for their career development.

To enable them to undertake the course in the context of other commitments, several participants took unpaid leave from their hospital positions to attend the course, with some undertaking locum positions to support themselves during this time. Others overcame this barrier by assisting surgeons in their private work; a source of funding available only to those in a surgical stream.

Financial costs associated with the course were regarded as a barrier to a greater degree for older students with more significant financial commitments who had to plan whether they could afford the cost of the course in the context of other living expenses. Others accepted the short-term financial hardship because they saw it is an important part of a longer-term career plan:

‘..I’m happy to make sacrifices because I have my eyes on the prize..money is a big thing. . ., you know, working as a junior doctor we work long, long hours and it’s not extremely well paid..’

In addition to financial costs, the significant time requirement of the course was also perceived as a barrier:

‘..for the amount of money you’re paying, you want to be able to be present for all the sessions. And if you can’t, then it’s arguably not worth your time..’

Time commitments also placed a strain upon participants’ personal lives. Some recognised that these difficulties were short-term and persevered despite the strain. Others were more concerned about the impact on relationships, missing out on events outside of work and study and a lack of work-life balance.

‘..I missed a lot of family things, things with the girlfriend, things with the family, cousins, you know uncles and aunts and stuff. .We had family things. . .my sisters came back from overseas. . . the rest. So there are a few things that I gave up, but again, I’m happy to do that. . . but it was trying to juggle the time. It was trying to get in here after work, get through the traffic, you know and sort of get back at night, study in addition to trying to exercise, sleep, eat, the rest. . . it’s been tricky..’

Some participants also struggled with time committed to the course and managing their employment. Juggling leave allocations and rostering presented challenges, but in addition several participants noted that their performance in their rotations was comprised. These participants were not seen as fully committed to their teams, and they perceived that this may have resulted in less than optimal references in a highly competitive field:

‘..I think that the time that I put into it detracted somewhat from my ability to stand out from other people, during that rotation..’

The practicalities of hospital rostering posed a further barrier to course attendance for some participants. Some described limited support from their employers to ensure that they had opportunities to attend the course through more flexible rostering from their employer. However, most participants described a lack of support from their employer in terms of rostering and time off as a barrier to course attendance.

Travel to attend the course also proved to be unexpectedly challenging and difficult for participants. They had not considered that there would be significant unscheduled overtime, making it difficult to arrive at their classes on time.

#### Course Structure

Participants’ overall responses to the course were uniformly positive and the high quality of teaching was valued. There was also a strong appreciation of the instructors’ enthusiasm for teaching anatomy.

Participants also held strong views about the structure of the course and the ways that it could be improved. Participants held a strong preference for having the course lectures available online in advance, so that they could be watched beforehand, thus allowing more time for dissection. Others requested either more teaching time or the opportunity to do more than one stream (in other words, to undertake detailed study and dissection of more than one region of the body). Participants did acknowledge that the limitations inherent in a single semester course (limited by specialty training scheme application deadlines, so that each year’s participants can have it on their CV in time), with limited time to accommodate these suggestions.

#### Unexpected Benefits

Course participants were interviewed towards the end of the course. As such, they had noted some benefits from participating that they had not anticipated, but still appreciated. Although not a key motivator for any of the participants, many reported that they valued the peer support and networking opportunities. Although many of the participants knew each other from medical school or from subsequent employment, this course allowed them to work together and reinforce relationships with their future colleagues.

Somewhat surprisingly, more than half of those interviewed reported that their professional confidence had significantly benefitted from attending the course. There are several possible explanations for this improvement, ranging from familiarity (many of the course instructors are senior specialists in prominent hospitals) through to a greater confidence in their own back-ground knowledge.

## Discussion

This research has explored the motivations for junior doctors to undertake further postgraduate training in anatomy as a precursor to entering speciality training and explored their experiences of attending one specific course. Several themes emerged from the research which have significant implications for the role of anatomy training in medical education.

The first major theme incorporated motivations for attending the Diploma. Candidates were advised by their near-peers, their supervisors and by the speciality training Colleges to which they aspired that attendance at the course, if not mandatory, was certainly advisable. Positions in speciality training programs are highly competitive and, as a result, candidates may feel pressure to undertake the course. Candidates may also feel insecure about their anatomy knowledge and perceive that they were not well-prepared by their undergraduate anatomy education ( Burgess and Ramsey-Stewart, 2014). The second major theme comprised participants’ perceived benefits from attending the course. This theme encompassed ideas about improved chances of selection into post-graduate training, and also included improvements in anatomy knowledge and acquiring a diverse range of skills that could not readily be achieved elsewhere. Finally, there was a theme which related to the price paid for these benefits, in terms of time, money and personal sacrifices. The opportunity costs, in terms of limitations on activities designed to benefit their specialty applications is a concern. The negative impact of undertaking the Diploma on work-life balance was also substantial.

There is a strong perception that completing the Diploma provides a significant advantage to a surgical career. Several training programs do offer formal credit towards acceptance for attending the Diploma, yet even those aiming for other specialty areas believed that the course would aid their efforts, whereas not completing the Diploma was perceived as a (potentially avoidable) deficit in preparing for a highly competitive selection process. As well as its role in the formal selection process, the participants identified other ways in which the program would aid their efforts to enter post-graduate training. Particularly important was impressing supervising specialists to achieve good references, which are crucial to access specialty training. Conversely, in some ways the Diploma limited participants’ opportunities to impress their supervisors and earn good references that are equally important in the struggle to enter post-graduate training.

Anatomical knowledge is assessed in specialty examinations by many training Colleges. Performance in these examinations determines whether a candidate will be granted entry to post- graduate training, how successfully they progress and whether they will successfully graduate. Many participants in this study perceived that their anatomical knowledge was poor and that they required additional training to ensure that their knowledge was adequate. This is in line with previous studies suggesting that many junior doctors have relatively poor anatomical skills gained through initial medical training ( Cottam, 1999; Gogalniceanu, O’Connor and Raftery, 2009). Thus, the course was perceived as an avenue to gain additional anatomical knowledge and skills and for participants to improve their examination performance relative to their peers.

The sacrifices made to complete the Diploma were regarded as well worthwhile if the candidate is accepted to and then successfully completes their desired post-graduate training program. It is worth noting that the current study did not aim to follow-up participants after they had completed the Diploma, nor to explore the outcomes for participants who completed earlier iterations of the Diploma. The findings of this study provide initial data on the motivations and expected benefits of undertaking the Diploma; however, a more formal evaluation of the benefits in terms of acceptance to speciality training programs and long-term career direction is required.

The findings of this study have several implications for the course developers. Participants were clear that the fees were a significant impediment to enrolling in and completing the course. At present, the course is over-subscribed, however future fee increases may significantly impact on potential candidates’ capacity to undertake the course. Because the course is resource-intensive, student numbers cannot be increased significantly without also expanding the resources required and therefore the costs of the program. Such difficulties are a potential threat to the ongoing viability of the program and participants’ perceptions that the program provided benefits that would justify the costs over the long-term.

A limitation of our study is the small sample size. Around 10% of the cohort that completed the Diploma in 2017 agreed to participate. Importantly, this is a single year of a course that has been running for over a decade. Experiences and motivations of other cohorts may vary. Participants may also have been reluctant to critique any aspects of the program until after the course was completed; however, none of the teachers involved in the course were part of the recruitment and interviewing process so that it is unlikely that participants would be concerned about honestly critiquing the program. Nonetheless, this research is a small exploratory study and future research might consider utilising the themes to undertake a broader review of participant perceptions of completing this course.

## Conclusion

The teaching of basic science, and particularly anatomy, in medical education has undergone remarkable changes over the last few years. The impact of these changes is unclear, importantly to ‘anatomical competence’ and the attainment of a level of knowledge that allows medical students to enter the workforce as a competent practitioner and to provide safe and effective medical practice. The group of junior doctors interviewed as part of this study recognised the limitations in their anatomy knowledge and actively sought additional training at significant financial and personal cost to themselves. This was counterbalanced by the perceived benefits to their career, and opportunity to enter a specialist training program, by completing additional study. Whether such benefits eventuate remains an area to be explored but this may be a moot finding because, as one participant highlighted:

‘..just because everyone does it, so you feel like you’re missing out if, if you’re not doing it..’

## Take Home Messages

• The literature on the perceptions of medical school anatomy teaching among those preparing to enter post-graduate training (i.e. towards a specialist qualification) is sparse.
• The experiences of junior doctors undertaking an intensive post-graduate anatomy training program fell into two broad categories: motivations for enrolling into the course and their actual experiences of the course.
• The primary motivation for enrolling was the perception that completion was mandatory for success in specialty training.
• The financial cost of the course, the unwillingness of employers to provide rostered study leave and the negative impact on work-life balance were identified as significant challenges.
• Whether the benefits of participating in training programs such as the one described in this study outweigh the significant personal and financial costs, remains to be explored.

## Notes On Contributors

Dr James Cameron a physician dual-qualified in internal medicine and emergency medicine. After serving as Director of Physician Training at a hospital he has extended his research interests to the preparation of junior doctors for higher qualifications. He also maintains an active clinical and educational practice

Dr Justin Bilszta is a Senior Lecturer with the Dept of Medical Education, Melbourne Medical School. He is involved in teaching and co-ordination of a number of academic programs within the Melbourne Medical School including the Doctor of Medicine (MD), Master of Clinical Education and Master of Clinical Research. Justin’s research interests include competency-based medical education, research methods pedagogy and influences on the patient-Dr relationship. ORCID - https://orcid.org/0000-0001-9578-591X

Dr Katharine Reid is the Director of Evaluation and Quality in the Department of Medical Education at The University of Melbourne. She is a psychologist with extensive experience in evaluation of educational programs and an interest in the assessment and selection of students for initial and specialist medical training. ORCID - https://orcid.org/0000-0002-4600-8847

Dr Chris Briggs was previously coordinator of the Graduate Diploma in Surgical Anatomy at the University of Melbourne. He continues to teach surgical trainees at James Cook University, Queensland Australia and tutor medical students at other institutions. His research interests include development of an interactive multimedia program, ‘Anatomedia’.
