# Supplementary material for: Motivations & Experiences of Postgraduate Anatomy Training
Source: MedEdPublish (2016). 2019 Mar 20;8:62. [Version 1] doi: 10.15694/mep.2019.000062.1 (PMC10712628; doi:10.15694/mep.2019.000062.1)
Supplement: Peer review history

# Reviewer report: Reviewer response for version 1

**DOI:** 10.21956/mep.19789.r29752

**Published:** 2019-03-31

**Authors:** Peter Dieter

**Affiliations:** 1 AMSE

This review has been migrated. The reviewer awarded 3 stars out of 5 This is an interesting article especially for anatomy. The article desribes a postgraduate training course to refresh anatomy knowledge.I have a few major suggestion how the article has to be improved substantially:- a similar survey should be done for biochemistry and physiology- the course should be free of costs (or much less expensive) for the trainees- all students should have the possibility to attend the course- I do not know if the students have been asked -before preparing the course- what deficits they might have from anatomy teaching and what should be taught in the postgraduate course

Reviewer Expertise:

NA

No decision status is available

# Reviewer report: Reviewer response for version 1

**DOI:** 10.21956/mep.19789.r29749

**Published:** 2019-03-27

**Authors:** Sateesh Babu Arja

**Affiliations:** 1 Avalon University School of Medicine

This review has been migrated. The reviewer awarded 4 stars out of 5 Thank you for asking me to review this article. It is a very well written paper and very clear to the reader. I certainly agree with authors that there has been less focus on teaching Anatomy in undergraduate medical education which can create a knowledge gap. And also we are moving from traditional cadaver dissection to modern teaching methods like 3-D dissection and Anatomage tables etc.. The one article that was published in Medical Teacher got my attention regarding Anatomy teaching "Do we really need cadavers anymore to learn anatomy in undergraduate medicine?"https://www.tandfonline.com/doi/abs/10.1080/0142159X.2018.1485884?journalCode=imte20I would like to appreciate authors Professors James Cameron, Justin Bilszta, Katharine J Reid, and Chris Briggs for taking up such an important topic qualitative analysis of Graduate Diploma in Surgical Anatomy. The research methodology is appropriate. But only my concern is 13 interviews look less. How did the authors come to the conclusion of themes? Is it based on grounded theory? If it is based on grounded theory, did saturation happen after 13 interviews and no more new information? It could have been informative if further details provided in the article. Thank you.

Reviewer Expertise:

NA

No decision status is available

# Reviewer report: Reviewer response for version 1

**DOI:** 10.21956/mep.19789.r29751

**Published:** 2019-03-24

**Authors:** BALAJI ARUMUGAM

**Affiliations:** 1 TAGORE MEDICAL COLLEGE AND HOSPITAL

This review has been migrated. The reviewer awarded 4 stars out of 5 Thanks for inviting me to review this article. • The qualitative research explored the experiences of junior doctors undertaking an intensive post-graduate anatomy training program fell into two broad categories: motivations for enrolling into the course and their actual experiences of the course. • The motivations for enrolling were career progression, expected benefits, Improvements in Knowledge and Exam Performance• Experiences of the course were expressed as Barriers to Attendance: Cost, Time, Leave & Rostering and Travel (Financial burden, time commitments, travel to attend the course), Course Structure (More teaching time, more online course lectures), Unexpected Benefits (professional confidence had significantly benefited from attending the course)• The study was well conducted, documented and analysed using the qualitative tool. • The interview method would have been detailed more like how the interview was initiated and conducted, who are interviewers, when was it planned and where was it conducted. Any specific reasons for the very poor response rate among the total of 115. • Similarly as a reviewer I am interested in knowing the details included in the supplementary file (interview guide)…• Overall the efforts of the team is well appreciated with the methodology of the study.

Reviewer Expertise:

NA

No decision status is available

# Reviewer report: Reviewer response for version 1

**DOI:** 10.21956/mep.19789.r29750

**Published:** 2019-03-21

**Authors:** Simonetta Ausoni

**Affiliations:** 1 Department of Biomedical Sciences- University of Padova- School of Medicine

This review has been migrated. The reviewer awarded 3 stars out of 5 I found the reading of this article very interesting. The qualitative study proposed by the authors examines a group of 13 junior medical doctors who have attended an intensive post-graduate training in anatomy. They were asked to express themselves in terms of motivations, advantages and benefits that could derive from having followed the course and finally considerations on technical aspects of course management (costs, time, organization). My first consideration is a general comment. I am absolutely convinced that going back to the basic sciences at the end of the medical program is crucial. The authors focused on anatomy, but we could also mention the training in molecular biomedicine, the analysis of big data, the study of embryology and other fields in growing expansion that often find little space in the medical programs, but would be highly effective if taken up again after graduation. The major problem is how to organize and economically support these activities in a medical school. In this regard, I would have liked to know more in detail how the whole anatomy course was organized during the semester. This piece of information, if provided exhaustively, would be useful for those who want to explore similar experience. Finally, I have to say that the initiative presented is of great interest, but the costs are really prohibitive. I think that the authors should figure out how to make it more affordable.

Reviewer Expertise:

NA

No decision status is available

# Reviewer report: Reviewer response for version 1

**DOI:** 10.21956/mep.19789.r29753

**Published:** 2019-03-20

**Authors:** Iain Keenan

**Affiliations:** 1 Newcastle University

This review has been migrated. The reviewer awarded 4 stars out of 5 This is an interesting and well-described article which provides important insights into the experiences and anatomical knowledge of postgraduate trainees undertaking an anatomy course. As the authors state, it would be valuable to perform a follow-up study to identify the impact of the course and to provide additional and longer-term data. Anatomical knowledge is likely to be essential to some extent in all specialities, so it is unfortunate that not all trainees have the opportunity to engage in such courses. Furthermore it will be important to explore not only what the impact of such postgraduate training has on future clinical practice and patient safety, but also to identify what may be able to be done during undergraduate anatomy education within medical programmes, which may in turn offset the need for such extensive and intensive postgraduate training courses.

Reviewer Expertise:

NA

No decision status is available
